# *Response to Dr. Roger’s letter*: further studies are necessary in order to conclude a causal association between the consumption of monosodium L-glutamate (MSG) and the prevalence of metabolic syndrome in the rural Thai population

**DOI:** 10.1186/1743-7075-10-10

**Published:** 2013-01-15

**Authors:** Tonkla Insawang, Carlo Selmi, Ubon Cha’on, M Eric Gershwin, Puangrat Yongvanit, Vitoon Prasongwattana

**Affiliations:** 1 Department of Biochemistry, Faculty of Medicine, Khon Kaen University, 40002, Khon Kaen, Thailand; 2 Division of Rheumatology, Allergy and Clinical Immunology, University of California at Davis School of Medicine, Davis, CA, USA; 3 Clinical Immunology, Humanitas Clinical and Research Center, Milan, Italy

**Keywords:** Monosodium glutamate, Intake, Metabolic syndrome

## Abstract

See related article: http://www.nutritionandmetabolism.com/content/10/1/14

## Background & discussion

We appreciate the helpful comments by Dr. Rogers but with all due respect there are a number of problematic issues with his remarks on our most recent publication [1]. Firstly, while we agree that nutritional epidemiology is extremely challenging, particularly on the scale and population herein, we must emphasize that the data speak by themselves. Indeed, the rigorous statistical approach argues for the potentially detrimental effects of monosodium glutamate (MSG) on this target population. Second, our observation is supported by recent animal data which, albeit with differences related to experimental models and dosage proportions, suggest a contribution of MSG to the worldwide epidemics of obesity, metabolic syndrome, diabetes and non-alcoholic steatohepatitis (NASH) [2-7]. It is well-established that obesity can be induced by an injection of MSG to new born rodents. Third, we note that data from one of the studies considered as negative by Dr. Rogers [8] reported a larger waist circumference, a crucial parameter of the metabolic syndrome, and an elevated prevalence of central obesity associated with the highest quartile of MSG intake, in an analysis similar to our approach. Moreover, results of a cohort study from the same group also suggested the potentially negative impact of MSG on human health; MSG intake was associated with a significant increase in systolic and diastolic blood pressure [9]. High blood pressure is one of the five criteria of ATPIII for the diagnosis of metabolic syndrome. Fourth, we agreed that there is inconsistency of the results of epidemiological studies on MSG intake and overweight which is always observed in epidemiological research field. So far, there are 3 studies showed the positive association and one study showed negative association of MSG intake and overweight. Fifth, the questionable points by Dr. Rogers are responded as follow;

1) The number of participants (n = 315) in Pethlert *et. al*, 2011[10] is not the same as Insawang *et. al*, 2012 (n = 349) because it is different project and time setting. Pethlert project was run after Insawang project. Not all participants in Insawang project were recruited into Pethlert project. Though the participants were the same group, some participants did not meet the requirements of Pethlert project such as unavailable during study period, did not return the informed consent, the levels of daily alcoholic drinking were exceeded the criteria of non-alcoholic fatty liver. Therefore, the number of Pethlert project is less than Insawang project.

2) Commercial MSG is always available and is not expensive for villagers to obtain it. Normally, only one person preparing food for family and that is the key person for MSG consumption in the family. We gave enough information to participants including key persons about the use and the objective of MSG measurement. Participants knew that it doesn’t matter how much the MSG left in the provided box, it will belong to them after 10-day of study. Therefore, it is unlikely that individuals use more MSG than usual because it is free.

3) Nowadays, it becomes a nuclear family in Thailand, including the area of study. Some families do not have children below 10 years old; however, some families have one or two children. Families usually prepare a special meal for young children by avoiding chili, MSG, salty or any spicy ingredients. Thus, it is not suitable to divide the MSG consumption in each family by number of all family members which may include infant, toddler, or pre-school children. Therefore, we excluded children under 10 for more reasonable calculation of individuals MSG consumption.

4) The association of the median and percentage values of the five criteria of ATP III individually and MSG intake were not observed in our study. This is probably due to the small number in sample size. However, when combined the metabolic disorders as a cluster, the association was found. Every 1-g of MSG intake significantly increased the risk of having metabolic syndrome with the odds ratio of 1.14. In other word, the estimated risk of having metabolic syndrome increased 1.14 fold for each gram of daily MSG consumption. If a person consumed MSG 5 g/day the estimated risk of having metabolic syndrome would be increased 5.7 fold. Therefore, it shall not classify as very week association.

5) It is barely to find non-MSG users in the area of study. Moreover, it is hard to identify a person with non-MSG user because if he/she eats out or buys food from retailer or restaurant; generally he/she cannot avoid MSG exposure. Therefore, non-MSG users were not included in our study.

Finally, we note that none of the investigators in our paper has any industrial or personal disclosure.

## Conclusion

We submit that MSG poses a host risk in susceptible individuals and that further data is needed to define those at risk.
